# Screening Depressive Symptoms and Incident Major Depressive Disorder Among Chinese Community Residents Using a Mobile App–Based Integrated Mental Health Care Model: Cohort Study

**DOI:** 10.2196/30907

**Published:** 2022-05-20

**Authors:** Huimin Zhang, Yuhua Liao, Xue Han, Beifang Fan, Yifeng Liu, Leanna M W Lui, Yena Lee, Mehala Subramaniapillai, Lingjiang Li, Lan Guo, Ciyong Lu, Roger S McIntyre

**Affiliations:** 1 Department of Medical Statistics and Epidemiology School of Public Health Sun Yat-sen University Guangzhou China; 2 Department of Psychiatry Shenzhen Nanshan Center for Chronic Disease Control Shenzhen China; 3 Mood Disorders Psychopharmacology Unit University Health Network Department of Psychiatry, University of Toronto Toronto, ON Canada; 4 Institute of Medical Science University of Toronto Toronto, ON Canada; 5 Department of Pharmacology University of Toronto Toronto, ON Canada; 6 Mental Health Institute of the Second Xiangya Hospital Central South University Changsha China

**Keywords:** screening, depressive symptoms, incident major depressive disorder, Chinese community residents, electronic-based integrated mental health care model

## Abstract

**Background:**

Depression is associated with significant morbidity and human capital costs globally. Early screening for depressive symptoms and timely depressive disorder case identification and intervention may improve health outcomes and cost-effectiveness among affected individuals. China’s public and academic communities have reached a consensus on the need to improve access to early screening, diagnosis, and treatment of depression.

**Objective:**

This study aims to estimate the screening prevalence and associated factors of subthreshold depressive symptoms among Chinese residents enrolled in the cohort study using a mobile app–based integrated mental health care model and investigate the 12-month incidence rate and related factors of major depressive disorder (MDD) among those with subthreshold depressive symptoms.

**Methods:**

Data were drawn from the Depression Cohort in China (DCC) study. A total of 4243 community residents aged 18 to 64 years living in Nanshan district, Shenzhen city, in Guangdong province, China, were encouraged to participate in the DCC study when visiting the participating primary health care centers, and 4066 (95.83%) residents who met the DCC study criteria were screened for subthreshold depressive symptoms using the Patient Health Questionnaire-9 at baseline. Of the 4066 screened residents, 3168 (77.91%) with subthreshold depressive symptoms were referred to hospitals to receive a psychiatric diagnosis of MDD within 12 months. Sleep duration, anxiety symptoms, well-being, insomnia symptoms, and resilience were also investigated. The diagnosis of MDD was provided by trained psychiatrists using the Mini-International Neuropsychiatric Interview. Univariate and multivariate logistic regression models were performed to explore the potential factors related to subthreshold depressive symptoms at baseline, and Cox proportional hazards models were performed to explore the potential factors related to incident MDD.

**Results:**

Anxiety symptoms (adjusted odds ratio [AOR] 1.63, 95% CI 1.42-1.87) and insomnia symptoms (AOR 1.13, 95% CI 1.05-1.22) were associated with an increased risk of subthreshold depressive symptoms, whereas well-being (AOR 0.93, 95% CI 0.87-0.99) was negatively associated with depressive symptoms. During the follow-up period, the 12-month incidence rate of MDD among participants with subthreshold depressive symptoms was 5.97% (189/3168). After incorporating all significant variables from the univariate analyses, the multivariate Cox proportional hazards model reported that a history of comorbidities (adjusted hazard ratio [AHR] 1.49, 95% CI 1.04-2.14) and anxiety symptoms (AHR 1.13, 95% CI 1.09-1.17) were independently associated with an increased risk of incident MDD. The 5-item World Health Organization Well-Being Index was associated with a decreased risk of incident MDD (AHR 0.90, 95% CI 0.86-0.94).

**Conclusions:**

Elevated anxiety symptoms and unfavorable general well-being were significantly associated with subthreshold depressive symptoms and incident MDD among Chinese residents in Shenzhen. Early screening for subthreshold depressive symptoms and related factors may be helpful for identifying populations at high risk of incident MDD.

## Introduction

### Background

Mental disorders account for significant illness-associated disability globally, and major depressive disorder (MDD, also called clinical depression) is one of the leading causes [1]. Moreover, MDD is highly prevalent, with high recurrence rates, nonrecovery, chronicity, and interepisodic dysfunction [2]. Along with staggering human costs, MDD exacts enormous individual and societal costs [3]. It is reported that MDD is closely associated with a loss of productivity and noticeable personal, social, and economic decline, thereby creating significant demands on patients, families, society, and service providers [4]. Moreover, at its worst, MDD can lead to increased risks of suicidal behavior (eg, suicidal ideation, suicide attempts, or even suicide death), and a large proportion of depressed individuals have suicidal ideation and suicide attempts [5-7]. According to the World Health Organization (WHO), depression affects >300 million people and represents a major contributor to the global burden of disease [8].

It is well documented that subthreshold depressive symptoms (ie, not meeting the minimum diagnostic threshold for a major depressive episode) could predispose and portend incident MDD, and previous evidence suggests that individuals manifesting subthreshold depressive symptoms have an approximately 2-fold higher risk of incident MDD than those without [9,10]. The increasing prevalence of MDD and its associated impact on human function is a national health priority for all countries, notably societies and health care systems in most low- and middle-income countries (LMIC) [8]. The additional challenge in LMIC is the observation that an estimated <10% of individuals with MDD in LMIC receive minimal treatment and support services [11]. Moreover, the gap in implementation of clinical practice guidelines for MDD is greater in LMIC than in high-income countries [12]. Early screening for subthreshold depressive symptoms has been reported to increase the likelihood of case identification among affected individuals, and a positive screen for subthreshold depressive symptoms is suggested to trigger an additional diagnostic assessment and, thus, improve future health [13]. The American Academy of Family Physicians and US Preventive Services Task Force recommend screening for depression in general adults [14]. Moreover, previous evidence indicated that early diagnosis and treatment of clinical depressive disorder might result in better outcomes [12] and seem to be more cost-effective [15]. In addition, early screening for depressive symptoms and depressive disorders has the potential to be effective. However, this had not been previously established.

During the past 30 years across China, rapid economic development and social change (eg, urbanization) have exposed citizens to changing factors. These rapid changes may be a determinant of adverse mental health problems (eg, subthreshold depressive symptoms and MDD) [16]. In keeping with this view, an increasing rate of depressive symptoms and mood disorders has been reported in China. For example, a recent national study using data from the China Mental Health Survey among Chinese adults reported that the weighted 12-month prevalence and lifetime prevalence of depressive disorder were 3.6% and 6.8%, respectively. Using the 2012 China Family Panel Studies data, a separate survey reported that 37.86% of the adult respondents experienced depressive symptoms [16]. Previous analyses from our group using data from the School-based Chinese Adolescents Health Survey reported a high prevalence (5.6% to 8.3%) of depressive symptoms among Chinese adolescents [17-19]. Moreover, a previous meta-analysis reported that the pooled lifetime prevalence of suicidal ideation and that of suicide attempts among patients with MDD in China were 53.1% and 23.7%, respectively [7]. However, access to mental health care in China remains constrained and needs to be improved [20].

### Cohort Study

Although 6 types of serious mental health disorders (including schizophrenia, schizoaffective disorder, persistent delusional disorder, bipolar disorder, mental disorders caused by epilepsy, and mental retardation accompanied by mental disorders) are recognized in community-based mental health management programs in China, MDD is not included. Previous evidence suggests that factors such as stigma-induced stress contribute to the unwillingness to seek professional help among individuals with depressive symptoms or MDD [21]. The increasing demand for mental health services and the shortage of psychiatrists in China have received the attention of Chinese policy makers and health care professionals [22]. Accordingly, China’s public and academic communities have reached a consensus on the need to improve access to early screening, diagnosis, and treatment of depression [23]. However, few studies have been conducted in China related to the screening and prevention of subthreshold depressive symptoms and MDD in community residents and the development of integrated mental health care models connecting primary, hospital, and community care divisions. Therefore, we performed this cohort study to estimate the screening prevalence and related factors of subthreshold depressive symptoms among community residents in Shenzhen, Guangdong province, China, using a self-developed mobile app–based integrated mental health care model and determine the 12-month incidence rate and related factors of incident MDD among individuals with depressive symptoms.

## Methods

### Study Design

Data were derived from the Depression Cohort in China (DCC) study (Chinese Clinical Trial Registry ChiCTR 1900022145), which is an ongoing longitudinal, population-based study for early identification, treatment, prevention, and management of subthreshold depressive symptoms and MDD [24]. We developed a Toronto-based Building Bridges to Integrate Care (BRIDGES) health care model to standardize the screening, detection, and treatment of individuals with subthreshold depressive symptoms or MDD in Nanshan district, Shenzhen, to meet the mental health needs of residents and their families [25]. With a population of approximately 2 million in an area of 185 km^2^, Nanshan is one of the most densely populated districts of Shenzhen.

### Ethics Approval

The study procedures were carried out in accordance with the Declaration of Helsinki. This study received ethics approval from the institutional review board of the School of Public Health, Sun Yat-sen University (L2017044), and the study protocol was approved by the ethics review boards of all the participating centers.

### BRIDGES Health Care Model

The DCC study used a BRIDGES health care model, which used the BRIDGES model, a project of the University of Toronto’s departments of medicine and family and community medicine, as a reference [25], to link mental health care delivery among primary health care centers, a general hospital, and a specialized mental health hospital in accordance with the health system in Nanshan. In the integrated health care model, individuals are screened at primary health care centers by general practitioners (GPs) at baseline and there is a referral gateway between primary health care centers and general and specialized mental health hospitals in the DCC study. Those who screen positive with subthreshold depressive symptoms at primary health care centers will be referred to general or specialized mental health hospitals to receive psychiatric diagnoses within a follow-up period of a maximum of 12 months. Participants referred through this gateway do not need to go through the hospital patient registration process and are given priority for care at the hospital. Considering that almost all GPs are not professional psychiatrists in China, psychiatrists from specialist hospitals trained GPs at primary health care centers to identify subthreshold depressive symptoms and provide usual care, referral, and follow-up for participants with these symptoms. Psychiatrists at hospitals provided outpatient or hospitalized care and education programs to patients diagnosed with MDD as well as follow-up, management, and referrals. Moreover, in the DCC study, project managers, who were public health physicians from general hospitals, supervised and ensured the quality of our integrated health care implementation process. Notably, in the DCC study, a mobile phone app, which included screening, referral, follow-up, and management functions, was developed and used by the GPs from the primary health care centers, psychiatrists from participating specialist hospitals, and project managers. Besides, the eligible participants at the primary care centers would be provided an account number by the GPs to access the app to complete the screening questionnaire and follow-up assessment when they visit the primary health care centers and hospitals in the corresponding study stages. The study process in the DCC study was performed through the self-developed mobile app, and it has been reported that this digital data collection mechanism may be a promising tool to collect data related to other diseases and risk factors [26].

### Participants

**Figure 1:**
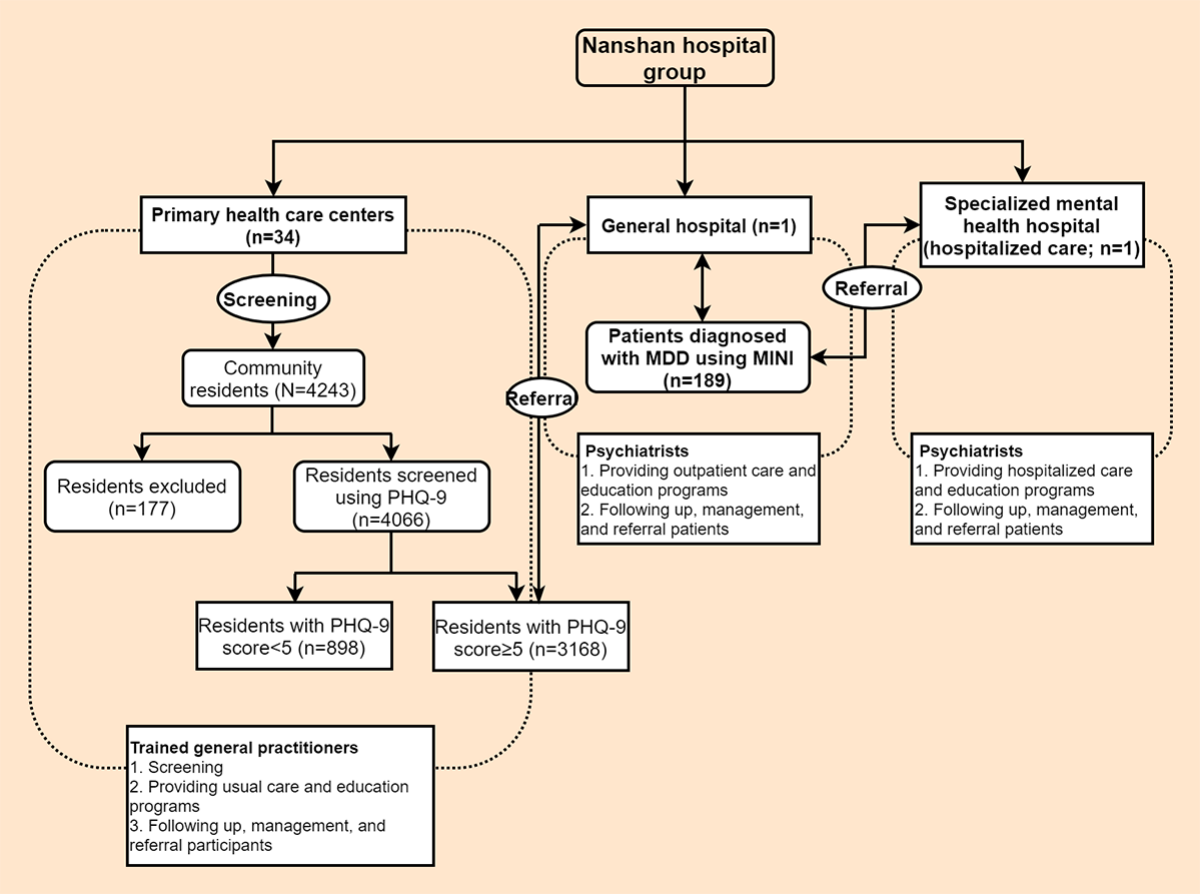
The integrated mental health care model in Nanshan, Shenzhen, in Guangdong province, China. MDD: major depressive disorder; MINI: Mini-International Neuropsychiatric Interview; PHQ-9: Patient Health Questionnaire-9.

The study data were drawn from an ongoing cohort study that began in early 2019 in which community residents are screened when they visit primary health care centers. Approximately 90,000 residents in Nanshan district walk through the doors of 34 primary health care centers a year. Among all the people visiting these centers, GPs selectively screen those who have mental health–related physical complaints (eg, sleep problems and chronic somatic pain) or are more likely to have mental health issues based on the GPs’ clinical experience and our study training. Our study aimed to screen individuals with subthreshold depressive symptoms and identify patients with MDD within limited medical resources and periods. Therefore, a total of 4243 community residents aged 18 to 64 years living in Nanshan were encouraged to participate in the DCC study when visiting the 34 participating primary health care centers at baseline, of which 177 (4.17%) residents were excluded (n=5, 2.8%, with incomplete information on depressive symptoms; n=133, 75.1%, with diagnostic depressive disorder; and n=39, 22%, with other psychiatric disorders; Figure 1), leaving 4066 (95.83%) residents who met the DCC study criteria and were screened for subthreshold depressive symptoms at baseline by the trained GPs at the participating primary health care centers. The DCC study exclusion criteria were as follows: (1) a diagnosis of current, or history of, depressive disorder, severe psychiatric disorder (ie, bipolar disorder, schizophrenia, schizoaffective mental disorder, paranoid mental disorder, mental disorders caused by epilepsy, or mental retardation), or alcohol or drug dependence disorder; (2) pregnant or perinatal women; (3) nonfluency in Mandarin; (4) inability to understand questionnaires or provide consent for themselves; (5) living outside the community; and (6) having a plan to leave Shenzhen within 12 months.

In this study, of the 4066 residents who met the study criteria, 3168 (77.91%) screened positive with subthreshold depressive symptoms at baseline at the primary health care centers and were referred to the general or specialized mental health hospitals to receive the psychiatric diagnoses within 12 months through the BRIDGES health care model. Psychiatric diagnoses were provided by the trained psychiatrists using the Mini-International Neuropsychiatric Interview (MINI; Diagnostic and Statistical Manual of Mental Disorders, Fourth Edition criteria). Among the patients with subthreshold depressive symptoms, 5.97% (189/3168) were first diagnosed with MDD during the follow-up period after the baseline screening (Figure 1). Written informed consent explaining the study purposes, processes, benefits, and risks was obtained from each participant.

### Measures

#### Subthreshold Depressive Symptoms

In the DCC study, subthreshold depressive symptoms were measured using the Patient Health Questionnaire-9 (PHQ-9), a widely used self-report measure in clinical and research settings that screens for depressive symptoms over the past 2 weeks [27]. The Cronbach *α* for PHQ-9 was .80 in this study. The PHQ-9 consists of 9 items, each addressing specific symptoms of depression during the past 2 weeks, and the scores for each item range from *0=not at all* to *3=nearly every day*, with a maximum score of 27. Higher scores were indicative of more severe depressive symptomatology. In this study, participants with a PHQ-9 score of ≥5 and without current, or a history of, depressive disorders were operationalized as having subthreshold depressive symptoms [24,28,29].

#### Ascertainment of Incident MDD

Participants with subthreshold depressive symptoms were referred to hospitals to receive the diagnosis of MDD within 12 months. The MINI, a Diagnostic and Statistical Manual of Mental Disorders, Fourth Edition, Text Revision–based validated structured diagnostic psychiatric interview, was used by psychiatrists to diagnose a current MDD and exclude other diagnoses [30].

#### Independent Variables

Sleep duration was assessed by the question, *How many hours do you usually sleep each day?* Anxiety symptoms were assessed by the Generalized Anxiety Disorder Scale-7 [31], which has been validated and extensively used in Chinese studies with satisfactory psychometric properties [32]. The Cronbach *α* was .92 with our sample. The 7 items’ total score ranges from 0 to 21, with higher scores indicating more severe anxiety symptomatology.

Well-being was measured using the 5-item World Health Organization Well-Being Index (WHO-5), which is a positively worded scale designed to measure the level of subjective well-being over the past 2 weeks on a 6-point scale ranging from 0 (not present) to 5 (constantly present), leading to a raw score ranging from 0 (absence of well-being) to 25 (maximal well-being) [33]. The Cronbach *α* for WHO-5 was .94 in this study.

Insomnia symptoms were assessed with the Insomnia Severity Index, which consists of 7 items, with each item scored from 0 to 4, for a maximum of 28 points. Higher scores represent greater insomnia levels [34], and the Cronbach *α* was .93 with our sample.

Adverse life events were measured using the Stressful Life Events Screening Questionnaire (SLESQ), which has been validated in Chinese studies [35], and the Cronbach *α* was .74 in this study. The SLESQ includes 12 items, each with a dichotomous response option (0=no and 1=yes). Higher scores reflect the experience of more adverse life events.

Resilience was measured using the Connor-Davidson Resilience Scale (CD-RISC), which comprises 25 items, with each rated on a 5-point scale ranging from 0 (not at all true) to 4 (true nearly all of the time). The Cronbach *α* for CD-RISC was .95 in this study. The CD-RISC yields a total resilience score ranging from 0 to 100, with higher scores indicating greater resilience [36].

The sociodemographic variables included in this study were age, sex (1=male and 2=female), ethnicity (1=Han Chinese and 2=Chinese minorities), education level (1=junior high school or below, 2=senior high school, and 3=college or above), living arrangement (1=living alone, 2=living with family, and 3=living with others), marital status (1=unmarried, 2=married, 3=divorced, and 4=widowed), lifetime smoking (assessed by the question, *Have you ever smoked a cigarette?* Responses were coded as 1=yes and 2=no) [37], onset age of smoking, lifetime drinking (assessed by the question, *Have you ever consumed at least one alcoholic drink of any kind?* Responses were coded as 1=yes and 2=no) [37-39], and onset age of drinking. History of comorbidities (including hypertension, diabetes, heart disease, stroke, thyroid disease, tumor, and others) were also collected (responses coded as 1=yes and 2=no).

### Statistical Analysis

Data were described as means (SDs) for normally distributed continuous variables and as medians (IQRs) for nonnormally distributed continuous variables, and frequency with percentage was used to describe categorical variables. Baseline characteristics were summarized according to baseline depressive symptoms. Mann-Whitney *U* tests or 2-tailed *t* tests for continuous variables and chi-square tests for categorical variables were conducted to compare baseline sample characteristics between participants with a PHQ-9 score of <5 and those with a PHQ score of ≥5, as appropriate. Univariate and multivariate logistic regression models were performed to explore the potential factors related to subthreshold depressive symptoms at baseline, and odds ratios (ORs) with 95% CIs were estimated. All the variables shown to be significantly associated with subthreshold depressive symptoms by the univariate logistic regression models were entered into the multivariate logistic regression models. Moreover, the 12-month incidence rate of MDD among participants with subthreshold depressive symptoms was calculated. Univariate and multivariate Cox proportional hazards models were performed to explore the potential factors related to incident MDD, and hazard ratios (HRs) with 95% CIs were also reported. All the variables shown to be significantly associated with incident MDD by the univariate Cox proportional hazards models were incorporated into the multivariate Cox proportional hazards models. In addition, we also explored the associations of observed risk factors with incident MDD using 3-knotted restricted cubic spline regression models, and the *P* values for the test of linearity hypotheses were reported. Moreover, regarding the *P* values calculated from the multivariate logistic regression models or multivariate Cox proportional hazards models, the false discovery rate was calculated to address the concern of potential type I errors and multiple hypotheses testing. The false discovery rate–adjusted *P* value was indicated by *q*, and the results were considered nominally significant when *q*<.10 [40]. The multiple imputation by chained equations method was applied for missing data [41]. All statistical analyses were conducted using Stata software (version 14.1; StataCorp LLC) and R statistical software (version 4.0.2; R Foundation for Statistical Computing). All statistical tests were 2-sided, and *P*<.05 was considered statistically significant.

## Results

### Baseline Sample Characteristics

The sample characteristics of all included participants at baseline are shown in Table 1. Among the 4066 participants, the mean age was 38.19 (SD 11.46) years, and 1541 (37.9%) were men; 576 (14.17%) reported an education level as junior high school or below; 484 (11.9%) reported living alone; 1080 (26.56%) reported lifetime smoking; 2424 (59.61%) reported lifetime drinking; and 974 (23.95%) reported having a history of comorbidities. The mean (SD) values of sleep duration, Generalized Anxiety Disorder Scale-7 score, WHO-5 score, Insomnia Severity Index score, SLESQ score, and CD-RISC score were 6.67 (4.68), 5.24 (4.88), 13.75 (6.05), 8.25 (6.54), 0.48 (1.15), and 59.05 (23.50), respectively. Of the 4066 participants, 3168 (77.91%) had a PHQ-9 score of ≥5. The differences between the groups with and without subthreshold depressive symptoms were not significant regarding the distribution of age, sex, ethnicity, lifetime smoking, and onset age of smoking. The characteristics of each item of the PHQ-9 among participants at baseline are presented in Multimedia Appendix 1.

**Table 1:** Baseline characteristics of participants according to subthreshold depressive symptom status (N=4066).

Variable		Participants				*P* value^a^
		Total	PHQ-9 score<5 (n=898)	PHQ-9 score≥5 (n=3168)		
Age (years), mean (SD)		38.19 (11.46)	38.35 (10.74)	38.14 (11.66)	.64	
**Sex, n (%)**						.67
	Male	1541 (37.9)	346 (38.53)	1195 (37.72)		
	Female	2525 (62.1)	552 (61.47)	1973 (62.28)		
**Ethnicity, n (%)**						.76
	Han Chinese	3922 (96.46)	869 (96.77)	3053 (96.37)		
	Chinese minorities	140 (3.44)	29 (3.23)	111 (3.50)		
	Missing	4 (0.1)	—^b^	—		
**Education level, n (%)**						<.001
	Junior high school or below	576 (14.17)	84 (9.35)	492 (15.53)		
	Senior high school	966 (23.76)	198 (22.05)	768 (24.24)		
	College or above	2516 (61.88)	615 (68.49)	1901 (60)		
	Missing	8 (0.2)	—	—		
**Living arrangement, n (%)**						<.001
	Living alone	484 (11.9)	83 (9.24)	401 (12.66)		
	Living with family	3018 (74.23)	723 (80.51)	2295 (72.44)		
	Living with others	447 (11)	69 (7.68)	378 (11.93)		
	Missing	117 (2.88)	—	—		
**Marital status, n (%)**						<.001
	Unmarried	997 (24.52)	178 (19.82)	819 (25.85)		
	Married	2927 (72)	704 (78.4)	2223 (70.17)		
	Divorced	117 (2.88)	13 (1.45)	104 (3.28)		
	Widowed	25 (0.61)	3 (0.33)	22 (0.69)		
Lifetime smoking (yes), n (%)		1080 (26.56)	225 (25.06)	855 (27)	.27	
Onset age of smoking (years), mean (SD)		19.50 (5.39)	19.52 (6.20)	19.49 (5.15)	.93	
Lifetime drinking (yes), n (%)		2424 (59.61)	566 (63.02)	1858 (58.65)	.02	
Onset age of drinking (years), mean (SD)		19.23 (4.93)	19.72 (4.90)	19.09 (4.94)	.008	
**History of comorbidities (yes), n (%)**		974 (23.95)	189 (21.05)	785 (24.78)	.02	
	Hypertension	396 (9.74)	84 (9.35)	312 (9.85)	.70	
	Diabetes	168 (4.13)	37 (4.12)	131 (4.14)	.99	
	Heart disease	45 (1.11)	8 (0.89)	37 (1.17)	.59	
	Stroke	13 (0.32)	1 (0.11)	12 (0.38)	.32	
	Thyroid disease	129 (3.17)	26 (2.9)	103 (3.25)	.67	
	Tumor	33 (0.81)	4 (0.45)	29 (0.91)	.21	
	Other	313 (7.7)	52 (5.79)	261 (8.24)	.02	
Sleep duration (hours per day), mean (SD)		6.67 (4.68)	7.25 (6.54)	6.50 (3.99)	<.001	
Anxiety symptoms, mean (SD)		5.24 (4.88)	1.37 (1.86)	6.34 (4.91)	<.001	
Adverse life events, mean (SD)		0.48 (1.15)	0.18 (0.65)	0.56 (1.24)	<.001	
Well-being, mean (SD)		13.75 (6.05)	12.45 (5.80)	18.35 (4.49)	<.001	
Insomnia symptoms, mean (SD)		8.25 (6.54)	3.45 (3.47)	9.61 (6.56)	<.001	
Resilience, mean (SD)		59.05 (23.50)	68.56 (26.36)	56.05 (21.69)	<.001	

^a^Mann-Whitney *U* test or 2-tailed *t* tests for continuous variables and chi-square tests for categorical variables were conducted to compare baseline sample characteristics between participants with and without subthreshold depressive symptoms, as appropriate.

^b^Not available.

### Factors Associated With Subthreshold Depressive Symptoms

Univariate logistic regression models reported that participants with education levels of junior high school or below (OR 1.26, 95% CI 1.05-1.50) and senior high school (OR 1.90, 95% CI 1.48-2.43) had higher risks of having subthreshold depressive symptoms than those with education level of college or above. Participants living with family (OR 0.66, 95% CI 0.51-0.84) were less likely to report subthreshold depressive symptoms than those living alone. Lifetime drinking (OR 1.20, 95% CI 1.03-1.40) and a history of comorbidities (OR 1.24, 95% CI 1.03-1.48) were positively associated with subthreshold depressive symptoms, as were anxiety symptoms (OR 1.78, 95% CI 1.70-1.87), insomnia symptoms (OR 1.30, 95% CI 1.27-1.32), and adverse life events (OR 1.73, 95% CI 1.52-1.96). The onset age of drinking (OR 0.98, 95% CI 0.96-0.99), sleep duration (OR 0.88, 95% CI 0.83-0.94), general well-being (OR 0.81, 95% CI 0.79-0.82), and resilience (OR 0.98, 95% CI 0.97-0.98) were negatively associated with subthreshold depressive symptoms.

After incorporating all significant variables from the univariate analyses, the multivariate logistic regression model demonstrated that only anxiety symptoms (adjusted OR [AOR] 1.63, 95% CI 1.42-1.87) and insomnia symptoms (AOR 1.13, 95% CI 1.05-1.22) were associated with an increased risk of subthreshold depressive symptoms. General well-being (AOR 0.93, 95% CI 0.87-0.99) was negatively associated with the risk of subthreshold depressive symptoms. Moreover, these factors were still significantly associated with subthreshold depressive symptoms after correcting for multiple testing (Table 2).

**Table 2:** Factors associated with subthreshold depressive symptoms among baseline participants.

Variable		Model 1^a^			Model 2^b^		
		OR^c^ (95% CI)	*P* value	Adjusted OR (95% CI)		*P* value	*q^d^*
Age (1-year increase)		1.00 (0.99-1.01)	.64	N/A^e^		N/A	N/A
Male (reference=female)		0.97 (0.83-1.13)	.66	N/A		N/A	N/A
Ethnicity (reference=Chinese minorities)		0.92 (0.61-1.39)	.69	N/A		N/A	N/A
**Education level (reference=college or above)**							
	Junior high school or below	1.26 (1.05-1.50)	.01	1.48 (0.57-3.88)		.43	.78
	Senior high school	1.90 (1.48-2.43)	<.001	1.13 (0.54-2.38)		.75	.89
**Living arrangement (reference=living alone)**							
	Living with family	0.66 (0.51-0.84)	.001	0.92 (0.30-2.84)		.89	.96
	Living with others	1.13 (0.80-1.61)	.48	0.54 (0.24-1.21)		.13	.39
**Marital status (reference=widowed)**							
	Unmarried	0.63 (0.19-2.12)	.45	N/A		N/A	N/A
	Married	0.43 (0.13-1.44)	.17	N/A		N/A	N/A
	Divorced	1.09 (0.29-4.15)	.90	N/A		N/A	N/A
Lifetime smoking (reference=no smoking)		1.11 (0.93-1.31)	.25	N/A		N/A	N/A
Onset age of smoking (1-year increase)		1.00 (0.97-1.03)	.93	N/A		N/A	N/A
Lifetime drinking (reference=no drinking)		1.20 (1.03-1.40)	.02	1.33 (0.39-4.59)		.65	.89
Onset age of drinking (1-year increase)		0.98 (0.96-0.99)	.009	0.96 (0.91-1.01)		.15	.39
History of comorbidities (reference=no comorbidities)		1.24 (1.03-1.48)	.02	1.26 (0.67-2.36)		.48	.78
Sleep duration (1-hour increase)		0.88 (0.83-0.94)	<.001	0.98 (0.94-1.02)		.33	.71
Anxiety symptoms (increase in score by 1)		1.78 (1.70-1.87)	<.001	1.63 (1.42-1.87)		<.001	<.001
Well-being (increase in score by 1)		0.81 (0.79-0.82)	<.001	0.93 (0.87-0.99)		.02	.09
Insomnia symptoms (increase in score by 1)		1.30 (1.27-1.32)	<.001	1.13 (1.05-1.22)		.001	.007
Adverse life events (increase in score by 1)		1.73 (1.52-1.96)	<.001	0.96 (0.74-1.24)		.76	.89
Resilience (increase in score by 1)		0.98 (0.97-0.98)	<.001	1.00 (0.99-1.01)		.99	.99

^a^The univariate logistic regression models were the unadjusted models.

^b^The multivariate logistic regression models incorporated all significant variables from the univariate analyses.

^c^OR: odds ratio.

^d^The false discovery rate–adjusted *P* value.

^e^N/A: not applicable.

### Factors Associated With Incident MDD Among Participants With Subthreshold Depressive Symptoms

Of the 3168 residents screened with subthreshold depressive symptoms at baseline, 189 (5.97%) met the first major depressive episode criterion between March 2019 and March 2020; the 12-month incidence rate of MDD among participants with subthreshold depressive symptoms was 5.97% (189/3168; Multimedia Appendix 2). Table 3 highlights the factors associated with incident MDD. The univariate Cox proportional hazards models reported that lifetime drinking (HR 1.51, 95% CI 1.10-2.06), a history of comorbidities (HR 2.05, 95% CI 1.44-2.91), anxiety symptoms (HR 1.24, 95% CI 1.21-1.27), insomnia symptoms (HR 1.15, 95% CI 1.13-1.18), and adverse life events (HR 1.37, 95% CI 1.28-1.47) were positively associated with elevated risks of incident MDD. General well-being (HR 0.80, 95% CI 0.78-0.83) and resilience (HR 0.98, 95% CI 0.97-0.99) were negatively associated with incident MDD. After incorporating all significant variables from the univariate analyses, the multivariate Cox proportional hazards models demonstrated that a history of comorbidities was independently associated with a 49% increased risk of incident MDD (adjusted HR [AHR] 1.49, 95% CI 1.04-2.14) and anxiety symptoms (AHR 1.13, 95% CI 1.09-1.17) were positively associated with incident MDD. General well-being was associated with a decreased risk of incident MDD (AHR 0.90, 95% CI 0.86-0.94). Moreover, these associations were still significant after correcting for multiple testing.

In addition, we used restricted cubic splines to flexibly model and visualize the associations of anxiety symptoms and well-being with the risk of incident MDD (Multimedia Appendix 3). A linear and positive association between the anxiety symptoms’ total score and risk of incident MDD was also found (*P* for nonlinearity=.90), and a nonlinear and negative association between the well-being scores and risk of incident MDD was observed (*P* for nonlinearity=.01).

**Table 3:** Factors associated with incident major depressive disorder among participants with subthreshold depressive symptoms.

Variable		Model 1^a^			Model 2^b^		
		HR^c^ (95% CI)	*P* value	Adjusted HR (95% CI)		*P* value	*q^d^*
Age (1-year increase)		1.00 (0.99-1.02)	.73	N/A^e^		N/A	N/A
Male (reference=female)		0.77 (0.56-1.05)	.10	N/A		N/A	N/A
Ethnicity (reference=Chinese minorities)		0.73 (0.36-1.50)	.40	N/A		N/A	N/A
**Education level (reference=college or above)**							
	Junior high school or below	0.86 (0.55-1.33)	.50	N/A		N/A	N/A
	Senior high school	1.00 (0.70-1.42)	.99	N/A		N/A	N/A
**Living arrangement (reference=living alone)**							
	Living with families	0.70 (0.46-1.06)	.01	N/A		N/A	N/A
	Living with others	1.00 (0.59-1.71)	.99	N/A		N/A	N/A
**Marital status (reference=widowed)**							
	Unmarried	0.48 (0.14-1.67)	.25	N/A		N/A	N/A
	Married	0.33 (0.08-1.12)	.07	N/A		N/A	N/A
	Divorced	0.73 (0.19-2.78)	.64	N/A		N/A	N/A
Lifetime smoking (reference=no smoking)		1.02 (0.73-1.40)	.93	N/A		N/A	N/A
Onset age of smoking (1-year increase)		1.00 (0.96-1.04)	.98	N/A		N/A	N/A
Lifetime drinking (reference=no drinking)		1.51 (1.10-2.06)	.01	0.98 (0.68-1.42)		.92	.92
Onset age of drinking (1-year increase)		0.99 (0.96-1.02)	0.39	N/A		N/A	N/A
History of comorbidities (reference=no comorbidities)		2.05 (1.44-2.91)	<.001	1.49 (1.04-2.14)		.03	.07
Sleep duration (1-hour increase)		1.01 (0.99-1.03)	.36	N/A		N/A	N/A
Anxiety symptoms (increase in score by 1)		1.24 (1.21-1.27)	<.001	1.13 (1.09-1.17)		<.001	<.001
Well-being (increase in score by 1)		0.80 (0.78-0.83)	<.001	0.90 (0.86-0.94)		<.001	<.001
Insomnia symptoms (increase in score by 1)		1.15 (1.13-1.18)	<.001	1.03 (1.00-1.07)		.05	.07
Adverse life events (increase in score by 1)		1.37 (1.28-1.47)	<.001	1.09 (1.00-1.19)		.05	.07
Resilience (increase in score by 1)		0.98 (0.97-0.99)	<.001	1.00 (0.99-1.02)		.67	.78

^a^The univariate logistic regression models were the unadjusted models.

^b^The multivariate logistic regression models incorporated all significant variables from the univariate analyses.

^c^HR: hazard ratio.

^d^The false discovery rate–adjusted *P* value.

^e^N/A: not applicable.

## Discussion

### Principal Findings

This prospective cohort study used a mobile app–based integrated mental health care model to link mental health care delivery among primary health care centers, a general hospital, and a mental health hospital in Nanshan, Shenzhen, and identify populations at high risk and factors contributing to elevated risks of subthreshold depressive symptoms and incident MDD among Chinese residents in Nanshan.

Of the 4066 community residents meeting the DCC study criteria, 3168 (77.91%) screened positive for subthreshold depressive symptoms at baseline in evaluations by GPs using the PHQ-9 at primary health care centers [42]. This rate was higher than the prevalence reported in a previous study among adults in mainland China aged ≥45 years between 2011 and 2012 (26%) [43] and in a study among community people with ≥1 chronic conditions in Hong Kong between 2009 and 2011 (17%) [29]. The aforementioned differences may be attributed to the use of different scales. Another explanation for these results may be that the rapid economic growth and social change in recent years were accompanied by a general increase in psychological pressure and stress in Shenzhen, one of the fastest-growing cities in China [44]. In addition, the higher screening rate observed in this study might also be explained by the successfully implemented integrated mental health care model. It means that the participants in the DCC study were not randomly selected, and they were invited for subthreshold depressive symptoms screening when they visited the participating primary health care centers for some physical health problems (eg, somatic and sleep problems), which were prevalent comorbidities in depressive symptoms and depressive disorder [45,46]. Moreover, most of the 4066 participants meeting the DCC study criteria had higher education levels (n=2516, 61.88%), were women (n=2525, 62.1%), and had lifetime drinking (n=2424, 59.61%) or a history of comorbidities (n=974, 23.95%), and these features had been reported to be possibly associated with depression development [29,47,48].

The univariate logistic regression models demonstrated that a lower level of education, lifetime drinking, a history of comorbidities, anxiety symptoms, insomnia symptoms, and adverse life events were positively associated with subthreshold depressive symptoms. In contrast, residents living with family, having an older onset age of drinking, having longer sleep duration, and having higher resilience were less likely to experience subthreshold depressive symptoms [49-52]. Our findings are consistent with the available evidence. The findings from the univariate analyses will be helpful for identifying community residents who may be at risk of subthreshold depressive symptoms. We should focus on high-risk groups who present with the aforementioned adverse characteristics. Although some previous evidence suggested that higher education levels were positively associated with an increased risk of depressive symptoms [47], others reported that depression was significantly more prevalent among those with a low education level [53]. These mixed results may be related to the different classification of education levels, the variety in sample characteristics (eg, age or biological gender), or the different socioeconomic environments. In this study, the observed finding of the unadjusted association between education level and subthreshold depressive symptoms may be related to the possibility that individuals with a lower education level in Shenzhen were more likely to struggle in their lives than those having an education level of college or above; therefore, they might be more likely to contend with emotional disturbance. Moreover, after incorporating all significant variables into the multivariate logistic regression models, the results showed that only anxiety and insomnia symptoms were significantly associated with an increased risk of subthreshold depressive symptoms, whereas general well-being was negatively associated with a risk of subthreshold depressive symptoms in this community sample. These findings may indicate that anxiety or insomnia symptoms are the core factors that influence the risk of subthreshold depressive symptoms. These symptoms may be the most important modifiable risk factors, and specific attention should be paid to populations experiencing anxiety or insomnia symptoms. It has been reported that anxiety and depressive symptoms overlap in various domains. For example, negative emotions and cognitive distortions may be the core causes or symptoms of anxiety and depressive symptoms, with differences in terms of severity [54]. Long-term anxiety symptoms are likely to lead to the onset of depressive symptoms [55]. A non–mutually exclusive explanation for the association between insomnia symptoms and depressive symptoms is sleep loss, resulting in cognitive and emotional impairments through the hyperactivity of the hypothalamic-pituitary-adrenal axis or increasing levels of inflammatory markers, which are possible common pathophysiological mechanisms of subthreshold depressive symptoms [56,57].

Regarding the situation of MDD in China, Huang et al [44] reported that the weighted 12-month prevalence of MDD was 3.6% among Chinese households between 2013 and 2015; Chen et al [58] found that the incidence of MDD was 4% among Chinese university students between 2007 and 2008. Taken together, a novel finding of this cohort study is the observed higher 12-month incidence rate of incident MDD among Chinese residents with subthreshold depressive symptoms (189/3168, 5.97%). The increased rate of incident MDD reported in this study may be attributed to the increased risks of developing a depressive disorder among individuals with subthreshold depressive symptoms compared with the general population [9,10]. Another explanation might be related to the fact that the follow-up period of some participants in this study occurred during the COVID-19 pandemic. The emergence of this global event has created an environment where many determinants of poor mental health are exacerbated, and depressive disorders had increased globally in 2020 because of the COVID-19 pandemic [59]. Our previous study using data from the DCC study also reported that the COVID-19 pandemic had a highly significant and negative impact on a population with subthreshold depressive symptoms [24]. In addition, this observed incidence rate of MDD might also indicate that implementing an app-based integrated mental health care model might be helpful for early detection of populations at high risk of the first episode of MDD.

Moreover, the univariate Cox proportional hazards models showed that lifetime drinking, a history of comorbidities, anxiety symptoms, insomnia symptoms, and adverse life events might predict an increased risk of incident MDD. A higher level of subjective well-being and resilience may predict a decreased risk of incident MDD. Findings from the univariate analyses may provide evidence for identifying populations at high risk for incident MDD and modifiable factors among individuals with subthreshold depressive symptoms. In addition, after accounting for all significant variables, the multivariate analyses indicated that only a history of comorbidities and anxiety symptoms were associated with an increased risk of incident MDD among populations with subthreshold depressive symptoms; a higher level of well-being significantly predicted decreased incident MDD risk. Furthermore, restricted cubic spline models demonstrated a linear and positive association between anxiety symptoms and the risk of incident MDD. Well-being was negatively associated with incident MDD in a nonlinear fashion, meaning that although individuals with lower general well-being might be at a higher risk of incident MDD, whereas those with a higher level of well-being might be less likely to develop MDD, the HR for incident MDD did not linearly decrease by the level of well-being. These findings suggest that recognizing and preventing individuals with a history of comorbidities or anxiety symptoms from developing MDD may be the focus of targeted intervention efforts, and a strategy of cultivating well-being might be a promising first step. A possible explanation for the association between a history of comorbidities and incident MDD is that depressive disorder is prevalent in patients with a physical disorder (particularly in those with severe conditions such as diabetes and stroke), and this comorbidity largely contributes to a poorer quality of life, worsening outcomes, higher medical costs, and more significant disability of the physical disorders [60]. Similar to the results of subthreshold depressive symptoms, a significant association between anxiety symptoms and incident MDD was examined. Comorbid anxiety symptoms are common in patients with depressive disorder, and it has been widely reported that these disorders may share common underlying pathophysiology [61]. Moreover, the observed protective effects of well-being on incident MDD may be explained by its effects on positive psychological functioning, capturing one’s level of positive life satisfaction and a sense of purpose in life [62]. Besides, a model promoted by Keyes [63] also implied that individuals experiencing many psychopathology symptoms were more likely to experience a low level of well-being and vice versa. Previous longitudinal studies have also shown the predictive value of well-being, specifically on depressive disorders [64]. Moreover, a previous study also provided possible evidence that supporting an eHealth intervention using a mobile app designed to improve the well-being of adults may be helpful for treating depressive symptomatology [65]. Hitherto, our findings suggest that targeted interventions to increase well-being may be effective in protecting against the risks of developing a depressive disorder.

### Limitations

Several limitations need to be addressed. First, only community residents in Nanshan, Shenzhen, were involved in this study; thus, the findings may not be fully generalizable to other regions. Second, the study sample was drawn from the DCC study, which recruited participants from the participating primary health care centers, and the study sample was not randomly selected. Therefore, this study had selection and sampling bias and the estimated screening prevalence of depressive symptoms among adults in Shenzhen might be overestimated. Third, we did not estimate the 12-month incidence rate of MDD among individuals without subthreshold depressive symptoms. Although it may be rare for individuals without depressive symptoms to exhibit a 12-month incidence of MDD, these populations may present different illness characteristics in the presence of MDD. Fourth, the variable of PHQ-9 was used as a dichotomous variable (ie, having or not having depressive symptoms) in this study, and we would like to use a different method to estimate the severity of depressive symptoms (ie, the polytomous variable of PHQ-9) in our future study. To reduce the risk of developing MDD, early screening of vulnerable populations and implementation of effective interventions targeting these symptoms are highly recommended. The strengths of this study included the longitudinal design, the large representative community-based sample, and the use of a clinically validated diagnostic interview (ie, MINI) to diagnose MDD.

### Conclusions

Using a mobile app–based integrated mental health care model, this study found that the screened prevalence of subthreshold depressive symptoms among community residents in Nanshan, Shenzhen, was high. More specifically, we reported that 5.97% (189/3168) of the individuals with subthreshold depressive symptoms developed MDD within 12 months. In addition, anxiety symptoms were associated with an increased risk of subthreshold depressive symptoms and incident MDD among the community residents, and the presence of a history of comorbidities may predict the elevated risk of incident MDD. Moreover, a higher level of general well-being might decrease the risks of subthreshold depressive symptoms and incident MDD. The results from our study highlight the following: (1) the 12-month incidence rate of MDD among populations with subthreshold depressive symptoms is high, and screening earlier on in the illness trajectory of individuals with subthreshold depressive symptoms and recognizing high-risk factors may lead to earlier detection and treatment of MDD; (2) more attention should be paid to vulnerable populations with adverse characteristics (eg, anxiety symptoms, insomnia symptoms, or adverse life events); and (3) the implementation of an integrated mental health care model (ie, linking community, primary health care centers, and hospitals) in China might be helpful for training GPs to provide essential mental health services, improving community residents’ access to mental health care as well as the timely referral and management of patients with MDD.

## Appendix

Multimedia Appendix 1 The characteristics of each item of the Patient Health Questionnaire-9 among participants at baseline.

Multimedia Appendix 2 Incidence of the first diagnosis of depressive disorder among participants with subthreshold depressive symptoms.

Multimedia Appendix 3 Restricted cubic spline models for the associations of (A) anxiety symptoms and (B) well-being with the risk of incident major depressive disorder.

## Abbreviations

BRIDGES: Building Bridges to Integrate Care
CD-RISC: Connor-Davidson Resilience Scale
DCC: Depression Cohort in China
GP: general practitioner
HR: hazard ratio
LMIC: low- and middle-income countries
MDD: major depressive disorder
MINI: Mini-International Neuropsychiatric Interview
OR: odds ratio
PHQ-9: Patient Health Questionnaire-9
SLESQ: Stressful Life Events Screening Questionnaire
WHO: World Health Organization
WHO-5: 5-item World Health Organization Well-Being Index
