# Supplementary material for: Screening Depressive Symptoms and Incident Major Depressive Disorder Among Chinese Community Residents Using a Mobile App–Based Integrated Mental Health Care Model: Cohort Study
Source: J Med Internet Res. 2022 May 20;24(5):e30907. doi: 10.2196/30907 (PMC9166637; doi:10.2196/30907)
Supplement: Multimedia Appendix 1 [file jmir_v24i5e30907_app1.docx]

| Multimedia Appendix 1. The characteristic of each item of PHQ-9 among participants at baseline (N=4066). | | | | |
| --- | --- | --- | --- | --- |
| PHQ-9 item | Participants | | | |
|  | Not at all | Several days | More than half of the days | Nearly every day |
| Little interest or pleasure in doing things, n (%) | 947 (23.3) | 1978 (48.6) | 774 (19.0) | 367 (9.0) |
| Feeling down, depressed, or hopeless, n (%) | 1376 (33.8) | 1905 (46.9) | 580 (14.3) | 205 (5.0) |
| Trouble falling or staying asleep, or sleeping too much, n (%) | 1198 (29.5) | 1391 (34.2) | 835 (20.5) | 642 (15.8) |
| Feeling tired or having little energy, n (%) | 757 (18.6) | 1975 (48.6) | 872 (21.4) | 462 (11.4) |
| Poor appetite or overeating, n (%) | 1520 (37.4) | 1622 (39.9) | 630 (15.5) | 294 (7.2) |
| Feeling bad about yourself — or that you are a failure or have let yourself or your family down, n (%) | 2204 (54.2) | 1271 (31.3) | 360 (8.9) | 231 (5.7) |
| Trouble concentrating on things, such as reading the newspaper or watching television, n (%) | 2162 (53.2) | 1260 (31.0) | 442 (10.9) | 202 (5.0) |
| Moving or speaking so slowly that other people could have noticed? Or so fidgety or restless that you have been moving a lot more than usual, n (%) | 2614 (64.3) | 988 (24.3) | 323 (7.9) | 141 (3.5) |
| Thoughts that you would be better off dead, or thoughts of hurting yourself in some way, n (%) | 3555 (87.4) | 370 (9.1) | 82 (2.0) | 59 (1.5) |
