# Supplementary material for: Screening Depressive Symptoms and Incident Major Depressive Disorder Among Chinese Community Residents Using a Mobile App–Based Integrated Mental Health Care Model: Cohort Study
Source: J Med Internet Res. 2022 May 20;24(5):e30907. doi: 10.2196/30907 (PMC9166637; doi:10.2196/30907)
Supplement: Multimedia Appendix 2 [file jmir_v24i5e30907_app2.docx]

| Multimedia Appendix 2. Incidence of the first diagnosis of depressive disorder among participants with subthreshold depressive symptoms (N=3,168). | | | |
| --- | --- | --- | --- |
| Outcome | | Cases, n | 12-months incidence rate, (%)^b^ |
| Depressive disorder^a^ | |  |  |
|  | Yes | 189 | 6.0 |
|  | No | 2979 | - |

PHQ-9, Patient Health Questionnaire-9.

a: Participants with subthreshold depressive symptoms were referred to hospitals to receive the diagnosis of incident depressive disorder within 12 months by psychiatrists using the Mini-International Neuropsychiatric Interview (MINI).
