# Supplementary material for: Screening Depressive Symptoms and Incident Major Depressive Disorder Among Chinese Community Residents Using a Mobile App–Based Integrated Mental Health Care Model: Cohort Study
Source: J Med Internet Res. 2022 May 20;24(5):e30907. doi: 10.2196/30907 (PMC9166637; doi:10.2196/30907)
Supplement: Multimedia Appendix 3 [file jmir_v24i5e30907_app3.docx]

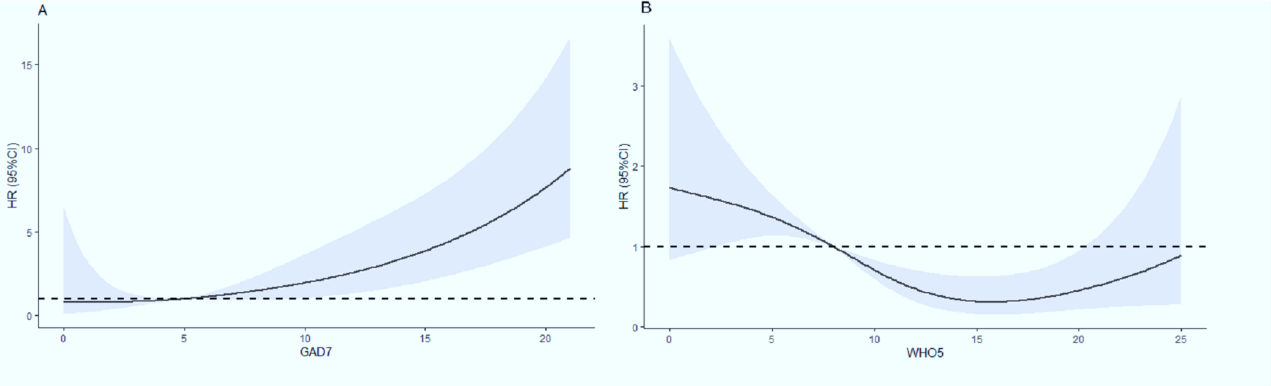


Multimedia Appendix 3**.** Restricted cubic splines models for the associations of anxiety symptoms (A) and well-being (B) with the risk of incident depressive disorder.
